# Opto-Mechanical Integrated Analysis of Micro-Vibration Effects on the Imaging Performance of a Precision Optical System

**DOI:** 10.3390/mi17050519

**Published:** 2026-04-24

**Authors:** Ruijing Liu, Zhen Liang, Yuying Zhang, Qingya Li

**Affiliations:** 1 College of Mechanical and Vehicle Engineering, Changchun University, Changchun 130022, China; liurj@ccu.edu.cn (R.L.); liangzhen1207@163.com (Z.L.); 2 College of Electronic and Information Engineering, Changchun University, Changchun 130022, China; zhangyy@ccu.edu.cn; 3 Changchun Institute of Optics, Fine Mechanics and Physics (CIOMP), Chinese Academy of Sciences, Changchun 130033, China

**Keywords:** micro-vibration, opto-mechanical integration analysis, precision optical system, micro-optics, image quality, reaction wheel, MOEMS

## Abstract

To explore the influence of reaction wheel perturbations on the image quality of a space optical telescope, a comprehensive dynamic model of a precision optical system was established, and an optical-mechanical integrated analysis approach was adopted to calculate the line-of-sight (LOS) error of the optical telescope under reaction wheel disturbances and determine the key mode that contributes the most significantly to the LOS error based on the entire satellite hierarchy. The rigid body displacements and mirror deformations generated by the optical reflector under reaction wheel perturbations were analyzed in synergy with the optical system to illuminate the impact of reaction wheel perturbations on the imaging quality of the optical imaging system. Finally, a satellite micro-vibration experiment was conducted, and the relative errors between the simulation and the experiment of the optical telescope’s object space axis of LOS error under key modes were 9.34% and 6.52% respectively, thereby validating the accuracy of the simulation analysis. The analysis outcomes offer direct engineering guidance for the structural layout and vibration isolation design of on-orbit optical satellites. The core innovations of this study are primarily manifested in three aspects: First, a full-link optomechanical integrated analysis framework is established, which synergistically accounts for the coupled effects of mirror rigid-body displacement and surface deformation on imaging performance, thereby addressing the limitations of single-factor analysis in existing research. Second, the framework is validated through satellite micro-vibration experiments, with the relative errors between simulation and experimental results both below 10%, ensuring the engineering reliability of the proposed method. Third, the scope of micro-vibration analysis is extended across scales from macroscopic space optical systems to micro/nano-scale precision optical devices. Beyond its application to space telescopes, this framework can be directly generalized to micro-optical systems sensitive to micro-vibrations, including augmented reality (AR) near-eye displays, microlithography objectives, and MOEMS-based micro-devices. The proposed framework is universal and can be directly extended to micro-optical systems such as MOEMS-based devices, near-eye display modules, and photonic crystal optomechanical systems, providing a standardized analytical approach for anti-vibration design in micro-system engineering.

## 1. Introduction

High-resolution optical systems serve as core equipment across diverse scientific and industrial domains, including remote sensing, astronomical observation, and precision manufacturing. A prevalent challenge spanning these fields lies in the escalating sensitivity of such high-precision systems to micro-vibrations, whose induced jitter can substantially degrade imaging performance. Whether for space-borne telescopes or ground-based microlithography equipment, comprehending and mitigating the effects of micro-vibrations is pivotal to achieving optimal optical system performance.

While micro-vibration-induced displacements are small in amplitude, they can exert severe adverse impacts. Regardless of the imaging modality employed, micro-vibrations may trigger rigid-body shifts and deformations of optical components within the system, leading to line-of-sight (LOS) errors and ultimately reducing the Modulation Transfer Function (MTF) of the optical imaging system [1,2]. Reaction wheels are among the most significant sources of micro-vibrations in orbiting satellites, as their high-speed rotation for attitude control generates broadband disturbances that directly couple into the optical system and degrade imaging performance.

Distinct from traditional analytical methods, optomechanical integrated analysis comprehensively accounts for the coupling between optical and mechanical fields, enabling unified and coordinated processing of multi-disciplinary data while overcoming the inherent limitations of conventional approaches. It stands as a critical tool for guiding the design of optomechanical structures and evaluating the performance of complex optical systems—ranging from large-scale telescopes to micro-optical devices [3]. The effects of mirror rigid-body displacement and surface deformation on MTF are typically analyzed via this integrated methodology [4].

Advancements in computer technology have driven significant progress in optomechanical integrated analysis techniques. Numerous scholars have leveraged MATLAB R2018b (MathWorks, Natick, MA, USA) to develop optomechanical interface programs for conducting integrated analyses of complex systems. For instance, Moon et al. [5] investigated the structural deformation of space telescopes under static and dynamic loads, assessing the impact of such deformation on optical systems using LOS error sensitivity equations. Building on this foundation, the Institute of Optics and Electronics, Chinese Academy of Sciences [6], and the Changchun Institute of Optics, Fine Mechanics and Physics, Chinese Academy of Sciences [7], developed an optomechanical interface program for data interaction, which employs Zernike polynomial fitting algorithms to evaluate optical system performance. Additionally, Lü Zhuang of Jilin University [8] utilized optomechanical integrated analysis to study micro-vibrations in space optical telescopes; however, this work only considered the effect of rigid-body displacement on LOS errors, neglecting the role of surface deformation, and its findings provided key evaluation criteria for system vibration isolation design.

Currently, the analysis of micro-vibration-induced degradation in optical system imaging quality has emerged as a research focus in the fields of space optics and micro-nano optics [9,10]. Existing studies predominantly concentrate on single scales: in macroscopic space optical systems, research has largely focused on single-factor analyses of either rigid-body displacement or surface deformation [5,8], with a lack of quantitative investigations into their coupled effects; in micro-nano-scale optical devices (e.g., MOEMS, AR near-eye display modules), micro-vibration analysis often relies on simplified models [1], and a full-link optomechanical integrated analysis framework that accounts for both mechanical disturbances and optical performance remains unestablished. Concurrently, verification methods in existing research are mostly simulation-based [6,7,9], with few studies integrating on-orbit physical experiments, resulting in insufficient measured data to support the engineering reliability of the methods. Furthermore, cross-scale adaptability research between micro-vibration analysis methods for macroscopic space optical systems and micro-nano optical devices remains a gap in the field—a gap that holds significant implications for advancing vibration-resistant design in micro-nano optical manufacturing.

These studies fully demonstrate that optomechanical integrated modeling, as a robust tool for predicting the performance of complex optical systems during the design phase, is growing in importance. This methodological trend is equally critical for the development of advanced micro-optical devices and systems, where the coupling between mechanical disturbances and optical performance becomes increasingly pronounced at micro-scales [1,10].

Optical-mechanical analysis is also applicable to micro-scale optical systems. Kim and Youk systematically quantified how micro-vibrations distort the edge spread function and alter MTF values in high-resolution electro-optical satellite payloads, establishing a theoretical foundation that applies across scales [11]. Zhang et al. designed a silicon-based photonic crystal cavity optomechanical system with an optical-mechanical coupling rate at the GHz/nm level, demonstrating the extreme sensitivity of such micro-optical systems to mechanical motion [12]. Wang et al. analyzed the impact of reaction wheel micro-vibrations on star sensor imaging quality and developed a dedicated micro-vibration simulator, confirming that space micro-vibrations severely degrade optical performance [13]. Lin et al. further investigated the impact of micro-vibration on the optical performance of an airborne camera, showing that micro-vibrations induce image jitter and reduce MTF [14]. Collectively, these studies underscore a pressing need for systematic opto-mechanical integration frameworks capable of predicting and mitigating vibration-induced performance degradation—from macroscopic telescopes to micro-optical devices.

In this paper, we propose an optomechanical integrated analysis framework to investigate the impact of micro-vibrations on optical system performance, using a reaction-wheel-excited space telescope as the case study. The core advantage of this framework lies in its simultaneous consideration of mirror rigid-body displacement and surface deformation, enabling dynamic full-link simulation from mechanical excitation to optical response. While validated on a macroscopic system, the framework is generalizable and can be directly extended to vibration sensitivity analysis and optimization of various precision optical systems, including near-eye display devices, microlithography objectives, and other micro-optical assemblies. By explicitly elucidating the quantitative coupling effect of micro-vibration perturbations on MTF, this paper addresses the gap in existing optomechanical integrated analysis research that overlooks the combined influence of mirror rigid-body displacement and surface deformation. Unlike prior literature focusing on macroscopic systems, the proposed framework features both cross-scale applicability and experimental verifiability. It not only enhances the accuracy of micro-vibration analysis for space optical telescopes but also provides a generalized technical pathway for vibration-resistant design of micro/nano-scale precision optical devices, laying a methodological foundation for performance prediction and optimization during the design phase of high-precision optical systems in the aerospace and micro-manufacturing fields.

This paper explicitly elucidates the quantitative coupling effect of micro-vibration perturbations on the modulation transfer function (MTF), thereby addressing the gap in existing optomechanical integrated analysis research that overlooks the combined influence of mirror rigid-body displacement and surface deformation. Distinct from prior studies in the literature that focus on macroscopic systems, the framework proposed herein features both cross-scale applicability and experimental verifiability. It not only improves the accuracy of micro-vibration analysis for space optical telescopes but also provides a generalized technical pathway for the vibration-resistant design of micro/nano-scale precision optical devices, laying a methodological foundation for performance prediction and optimization during the design phase of high-precision optical systems in the aerospace and micro-manufacturing fields.

## 2. Theory

### 2.1. The Effect of Mirror Movement on Image Shift

The vibration of the mirror across various degrees of freedom exerts distinct effects on the image shift within the optical system. Taking a spherical mirror as an example, let us denote the radius of the spherical mirror as r and the height of incidence for the parallel beam as h [6].

**Figure 1:**
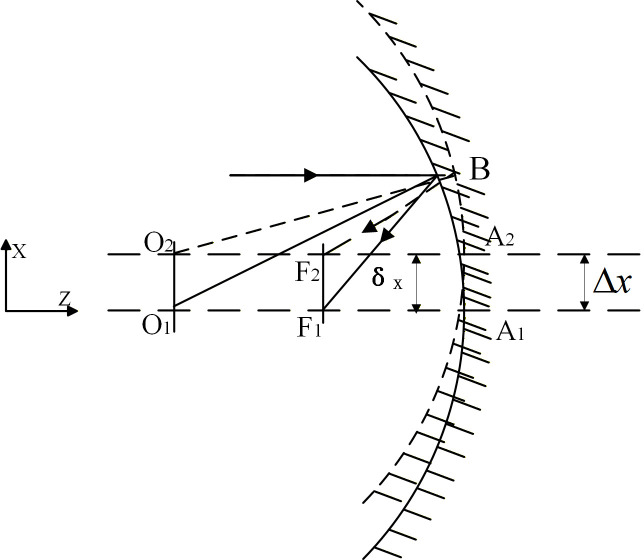
The decenter of the spherical mirror in the X direction, where ∆x is the translation of the spherical mirror in the X direction, and $\delta_{x}$ is the amount of image shift caused by the translation of the spherical mirror along the X direction.

**Figure 2:**
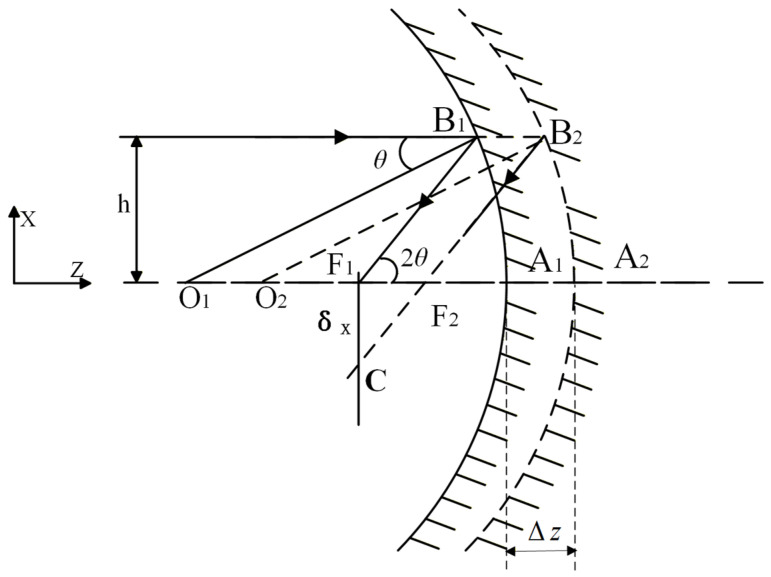
The despace of the spherical mirror in the Z direction.

**Figure 3:**
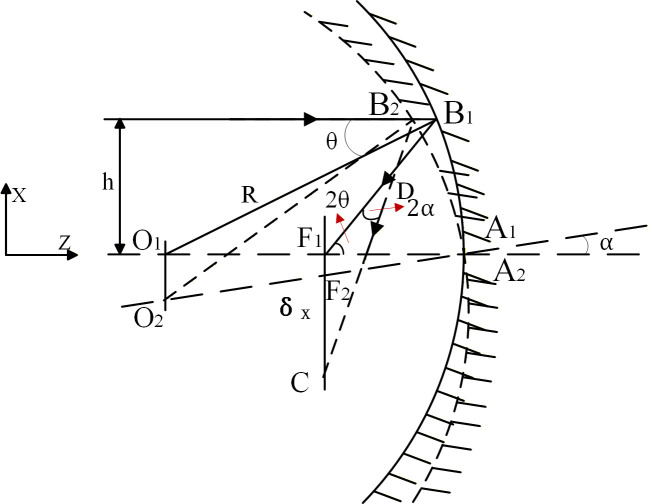
The spherical mirror tilts around the X axis.

As illustrated in Figure 1, Figure 2 and Figure 3 (schematic diagrams not drawn to scale). In Figure 1, when the spherical mirror undergoes translation along the x-direction (x-decenter), the focal point (F) of the spherical mirror is located on the line connecting the center of curvature (O) and the vertex (A) of the spherical mirror, and is the midpoint of the two points (i.e., f = R/2, where f is the focal length and R is the radius of curvature OA), in accordance with the geometric imaging principle of spherical mirrors.

For a general space optical telescope, the optical system exhibits symmetry in both the OX and OY directions. Consequently, the image shift resulting from translation along the Y direction should be equivalent to that caused by translation along the X direction. Analysis indicates that the decentering of the optical mirror along the X and Y axes due to micro-vibrations primarily leads to a corresponding shift of the image point in the same direction on the image plane. This conclusion is based on the geometric optics principle of spherical mirrors and the quantitative decenter-to-image-shift relationship derived below (i.e., the linear correlation between Δx/Δy and δx/δy). It should be noted that, in general, mirror decentering may also induce higher-order aberrations such as astigmatism in addition to image translation. However, this study focuses on the following specific conditions: (1) paraxial rays (incident height h satisfies the paraxial approximation); (2) small-amplitude micro-vibrations (maximum rigid-body displacement of mirrors on the order of 10^−3^ mm, see Section 3.2); and (3) near-ideal spherical mirror surfaces (RMS figure error < 10^−8^ mm, see Section 3.2). Under these conditions, the image translation caused by rigid-body decentering dominates, and the contribution of decenter-induced astigmatism to image quality is far smaller than that of the translational effect, thus not being a critical factor affecting the system MTF. Therefore, focusing on the image shift due to decentering while neglecting higher-order aberrations such as astigmatism is a reasonable and engineering-acceptable simplification in the opto-mechanical integrated analysis presented in this paper.

When the spherical reflector undergoes translation along the optical axis (Z direction), both its spherical center and focal point experience a corresponding displacement. As illustrated in Figure 2, this results in an image shift denoted as.

(1) θ≈sinθ≈tanθ=hR

(2) δx=F1C=Δztan2θ=ΔzR2h

where ∆z is the translation of the spherical mirror along the Z-direction, $\delta_{x}\;$ is the image shift caused by the translation of the spherical mirror along the Z-direction, and θ is the angle of incidence.

The image shift $\delta_{z}$ resulting from the translation of the spherical mirror along the Z-direction is linearly correlated with the offset ∆z in that same direction. The value of $\delta_{z}$ is influenced by both the radius of curvature R of the mirror and the incident height h of the sphere. An offset along the *Z*-axis leads to defocusing, which increases the size of the diffuse spot at the image point.

When the spherical mirror rotates (X-tilted) around the X axis at an angle ɑ. As shown in Figure 3, the angle of rotation of the reflected light is 2ɑ. When the angle of rotation of the reflector caused by micro-vibration is small, the incident point B1 and B2 of the near-axis light can be approximated as the same point, so F1B1≈F1B2=O1F1=R2. It should be noted that the above relation is derived under the paraxial approximation, i.e., the incident ray height h is much smaller than the radius of curvature R of the spherical mirror (h ≪ R), and the tilt angle ɑ is small (ɑ ≪ 1 rad). Under this condition, the change in the reflection point position can be neglected, leading to a simplified linear relationship. For the general case with h > 0, a correction term involving h would be required. However, given the extremely small amplitude of micro-vibrations in this study (see the rigid-body displacement magnitudes in Section 3.2) and the fact that the optical system satisfies the paraxial condition, the paraxial approximation is engineering acceptable.

(3) $$∠F_{1}F_{2}C=∠B_{1}F_{1}F_{2}+∠F_{1}B_{1}F_{2}=2\alpha +2\theta$$

According to the law of reflection and the geometric relations presented above, the tilt angle α of the spherical mirror about the *X*-axis results in a deflection angle of 2α for the reflected ray. By employing the paraxial approximation and the focal length relationship of the spherical mirror f = R/2, the linear relationship between the mirror tilt angle and the image shift δ on the focal plane can be established as: δ ≈ 2αf. Substituting f = R/2 into the above expression yields δ ≈ αR. On this basis, Formulas (4) and (5) are derived under the small-angle and paraxial conditions adopted in this study.

(4) F1C≈F1B1⋅sin∠F1B1F2=R2⋅2α=Rα

(5) $$\delta_{x}\approx \alpha \cdot R\cdot \tan\left[2\left(\theta +\alpha \right)\right]$$

The optical element tilts around the X and Y axes, thereby altering both the actual image plane and the ideal focal plane. The image captured by the detector represents a projection of the ideal image point. The shift in the image caused by the optical element is directly correlated with both the radius of curvature of the spherical mirror and its angle of rotational inclination.

For spherical mirrors, the mirrors are rotationally symmetric about the *Z*-axis, so tilting the mirror about the Z-direction has little effect on the optical system.

### 2.2. The Analysis of Line-of-Sight Error

**Figure 4:**
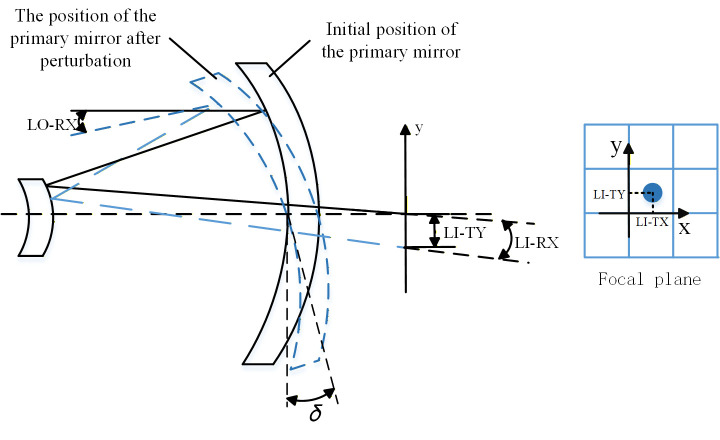
Schematic diagram of line-of-sight error. The black solid circle in the focal plane represents the image point; the vertical and horizontal arrows indicate the LI-TY (Y-direction) and LI-RX (X-direction) on the image plane, respectively.

The micro-vibrations of the space telescope structure, induced by the vibrations from the reaction wheels on the satellite, directly lead to both rigid body displacement and elastic deformation of the optical components within the space telescope. This phenomenon results in line-of-sight vibrations, characterized by lateral movements of optical image points on the image plane, as illustrated in Figure 4. Consequently, this degradation affects the modulation transfer function (MTF) of the optical imaging system, making visual axis error a significant metric for assessing imaging quality in optical systems [7].

Figure 4 presents a schematic diagram illustrating the sensitivity calculation of the Line-of-Sight (LOS) error. The optical reflector is influenced by micro-vibrations, resulting in a certain transfer angle, denoted as δ. Consequently, the eccentric LOS error in image space is represented as LI-TX/LI-TY, which corresponds to the lateral displacement of the image point within the detector plane. Additionally, the angular apparent LOS error is expressed as LI-RX/LI-RY, indicating the change in angle of the image point on the detector plane. In object space, the LOS error can be quantified as LO-RX/LI-RY; this reflects the angular variation in an object’s position at infinity. The sensitivity of LOS errors can be computed using Equations (6)–(11). These sensitivities may be integrated to develop a linear optical model for predicting apparent LOS errors. SigFit 2012b is a professional opto-mechanical integration software package. The basic procedure for calculating the LOS sensitivity matrices is as follows: First, the nodal displacements of each optical surface obtained from finite element analysis are imported into Sig Fit. Next, through Zernike polynomial fitting, the nodal displacements are decomposed into rigid-body motion (six degrees of freedom: translations and rotations) and surface deformation. Finally, using ray tracing algorithms, the lateral image displacement (LI-TX/LI-TY) and angular changes (LI-RX/LI-RY, LO-RX/LO-RY) induced by a unit rigid-body displacement are computed, thereby constructing the sensitivity matrices.

(6) $$\mathrm{LI}\text{-}\mathrm{TX}={\left[L\right]}_{\mathrm{LI}\text{-}\mathrm{TX}}\delta_{\mathrm{optic}}$$

(7) $$\mathrm{LI}\text{-}\mathrm{TY}={\left[L\right]}_{\mathrm{LI}\text{-}\mathrm{TY}}\delta_{\mathrm{optic}}$$

(8) $$\mathrm{LI}\text{-}\mathrm{RX}={\left[L\right]}_{\mathrm{LI}\text{-}\mathrm{RX}}\delta_{\mathrm{optic}}$$

(9) $$\mathrm{LI}\text{-}\mathrm{RY}={\left[L\right]}_{\mathrm{LI}\text{-}\mathrm{RY}}\delta_{\mathrm{optic}}$$

(10) $$\mathrm{LO}\text{-}\mathrm{RX}={\left[L\right]}_{\mathrm{LO}\text{-}\mathrm{RX}}\delta_{\mathrm{optic}}$$

(11) $$\mathrm{LO}\text{-}\mathrm{RY}={\left[L\right]}_{\mathrm{LO}\text{-}\mathrm{RY}}\delta_{\mathrm{optic}}$$

where [L]LI-TX, [L]LI-TY, [L]LI-RX, [L]LI-RY, [L]LO-RX and [L]LO-RY are the LOS sensitivity matrices calculated by Sig Fit as described above, and $\delta_{\mathrm{optic}}$ is the rigid-body motion matrix of 6 degrees of freedom for each optical element. The above calculations relate the rigid body motion of the optical elements to the object space or image space.

### 2.3. Analysis of Optical Modulation Function of Optical Imaging System Under Reaction Wheel Disturbance

The micro-vibrations generated during the operation of the reaction wheel in orbit, transmitted through the mechanical structure will cause image lateral motion (vibrations generated in the direction perpendicular to the optical axis, that is, X/Y direction) between the object and the focal plane sensor, making the image brightness in the detector plane blurred and a certain degree of loss of optical resolution 8.

**Figure 5:**
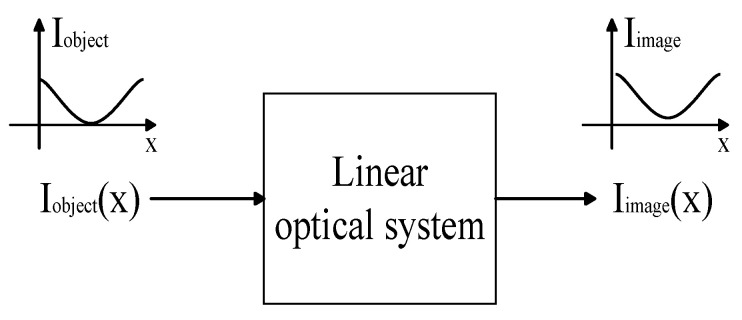
Linear Optical System.

For a linear optical system, as shown in Figure 5, an object whose luminance varies according to the sinusoidal law $I_{\mathrm{object}}\left(x\right)$ with spatial frequency f, after imaging by the optical system, the image luminance remains in the sinusoidal form $I_{\mathrm{image}}\left(x\right)$ that varies according to the same spatial frequency, but the contrast generally decreases. The contrast (modulation) is defined as the maximum brightness difference of the normalized image [9].

(12) $$K=\frac{I_{\max}-I_{\min}}{I_{\max}{+I}_{\min}}$$

(13) $$\mathrm{MTF}\left(f\right)=\frac{K_{\mathrm{image}}\left(f\right)}{K_{\mathrm{object}}\left(f\right)}$$

By applying the weighting function wd to the PSD response of the Line-of-Sight (LOS) error, we can decompose the LOS error into two components: a jitter term and a drift term. Given that the jitter term exerts a significantly greater influence on the Modulation Transfer Function (MTF) compared to the drift term, our analysis primarily focuses on the jitter component. The corresponding jitter weight function is articulated in Equation (14) [10].

(14) $$W_{d}=\frac{1-2\left(1-\cos\left(2\pi fT\right)\right)}{(2\pi fT{)}^{2}}$$

where *f* is the spatial frequency and T is the integration time.

The RMS value of the jitter term is the square root of the enclosed area under the jitter PSD response curve, as shown in Equation (15).

(15) $$\Delta_{\mathrm{jitter}\_\mathrm{rms}}=\sqrt{\int_{0}^{\infty }W_{d}\left(f\right)\Delta_{\mathrm{PSD}}\left(f\right)df}$$

(16) $${\mathrm{MTF}}_{\mathrm{jitter}\_\mathrm{random}}\left(f\right)=e^{-{2\pi }^{2}\Delta_{\mathrm{jitter}\_\mathrm{rms}}^{2}f^{2}}$$

(17) $${\mathrm{MTF}}_{\mathrm{Net}}{=\mathrm{MTF}}_{\mathrm{Nominal}}\times {\mathrm{MTF}}_{\mathrm{jitter}\_\mathrm{random}}$$

where ${\mathrm{MTF}}_{\mathrm{jitter}\text{-}\mathrm{random}}$ is the influence factor, ${\mathrm{MTF}}_{\mathrm{Net}}$ is under the influence of vibration, and ${\mathrm{MTF}}_{\mathrm{Nominal}}$ is the initial design.

## 3. Optical and Mechanical Integrated Simulation Analysis

**Figure 6:**
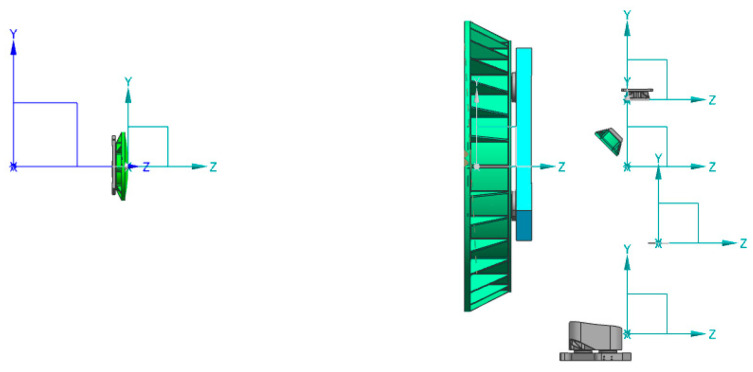
Establishment of the local coordinates of the vertex of the optical mirror.

**Figure 7:**
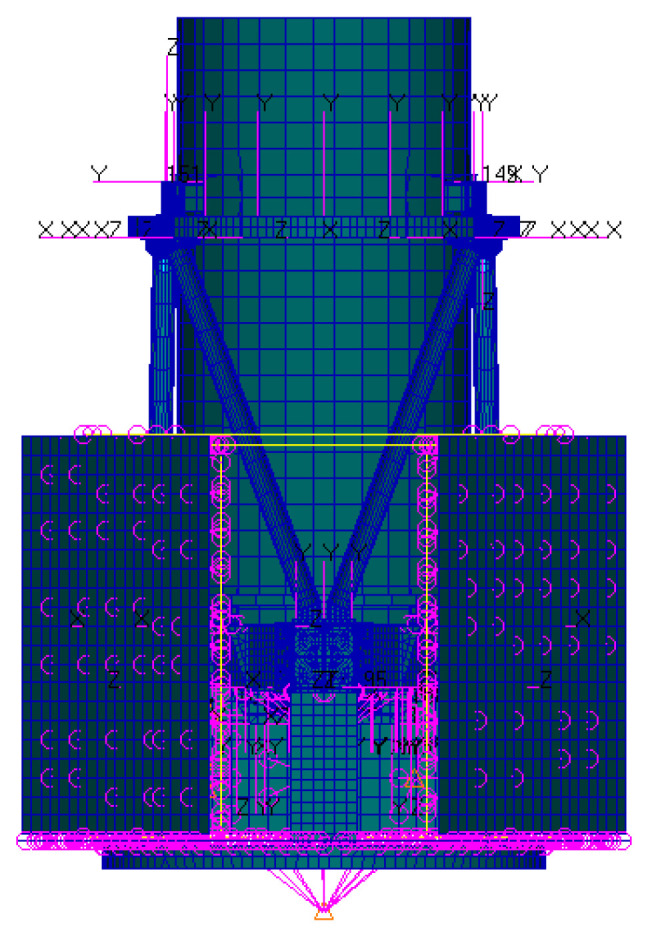
The finite element model of satellite.

To assess the influence of mechanical disturbances on the image quality of optical systems, it is indispensable to guarantee the congruence in units, geometry, and coordinate systems between the mechanical model and the optical system model. In coaxial optical systems, the apex of the optical mirror coincides with its geometric center. Nevertheless, for off-axis systems, the apex position is eccentric. To maintain the coordinate consistency between the two models, the following approaches can be adopted: (1) defining a virtual surface and a coordinate breakpoint within the optical model that aligns with the mechanical model and is located at the physical center of the matrix; (2) integrating a rigid connection into the finite element model to correlate the average rigid body motion of the optical surface with the apex position; (3) establishing a local coordinate system in the mechanical model based on the coordinates derived from the mirror apex of the optical system. In this paper, method (3) is selected to establish the connection between the optical surface and the mirror apex in the mechanical model, as depicted in Figure 6. Based on the finite element theory, the entire star finite element model was constructed using Hypermesh 2017 (Altair Engineering, Inc., Troy, MI, USA) software, as presented in Figure 7.

### 3.1. The Analysis of Line-of-Sight Error

**Figure 8:**
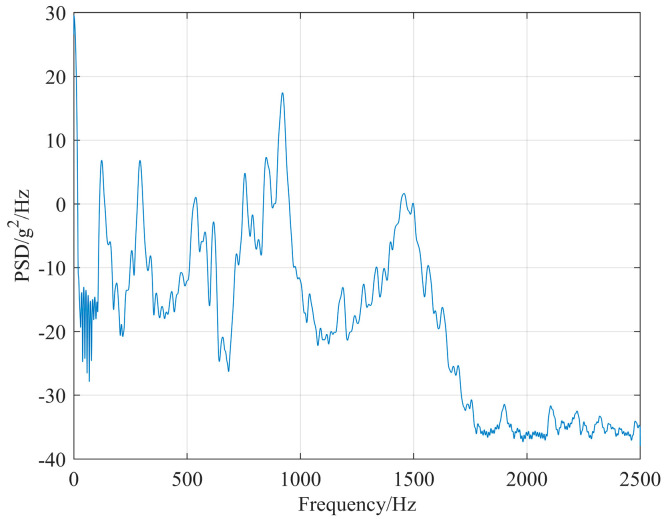
Y-direction reaction wheel micro-vibration PSD.

**Figure 9:**
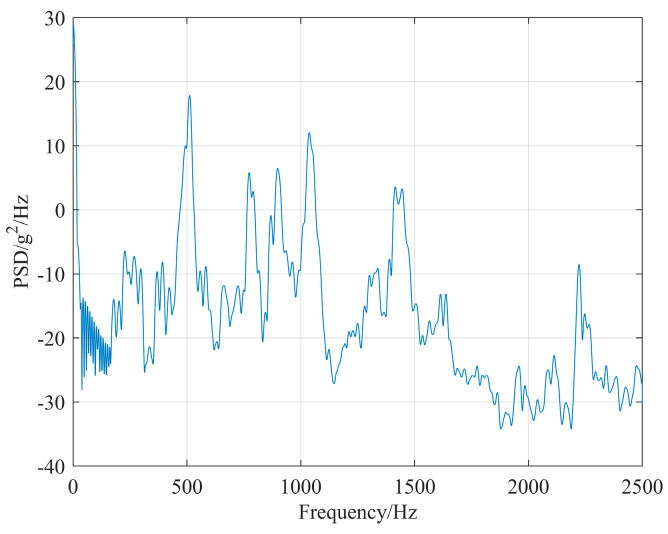
Z-direction reaction wheel micro-vibration PSD.

Since micro-vibrations encompass a broad spectrum, with vibration frequencies ranging from 0 to 10 kHz, and considering that the satellite operates in a free state characterized by a prolonged vibration decay period during its orbital mission, the perturbations generated by the reaction wheel are typically represented as power spectral density (PSD) inputs for telescope perturbation [10]. The attitude control strategy for the reaction wheels during the operational phase of this optical satellite involves operating the Y-direction reaction wheel at 600 rpm and the Z-direction reaction wheel at 1200 rpm for most of the time. Perturbation experiments were conducted on both Y- and Z-direction reaction wheels to collect perturbation data. This data was subsequently processed using MATLAB and utilized as input for perturbation PSD, as illustrated in Figure 8 and Figure 9.

The simulation results indicate that the primary modes contributing to the line-of-sight (LOS) error in this high-resolution optical satellite system are the 8th and 9th order modes. The contribution percentage is defined as the ratio of the LOS response magnitude contributed by a given mode to the total LOS response across all modes. Taking the object space angular displacement (LO-RV) as a case study, it is observed that the contribution percentages of the 8th and 9th order modes to LO-RV are 53.737% and 36.788%, respectively. Given that contributions from other order modes are comparatively minor, they have not been included here; instead, only the 4th through 10th order modes are presented in Table 1 for comparison of trends before and after observation. Note that the 4th to 6th order modes are marked with a frequency of 0.00 Hz, which represents the rigid-body modes of the satellite optical system (zero-frequency rigid-body motion), reflecting the low-frequency rigid-body translation/tilt characteristics of the system without elastic deformation.

The LOS error in image space is expressed as the lateral movement of the image point on the image plane LI-TX/LI-TY, and in object space is expressed as the change in the position angle of the object point at infinity LO-RX/LO-RY. The corresponding LOS errors in object space for each mode are listed in Table 2. These results further confirm that the 8th and 9th modes dominate the LOS response, with the 8th mode contributing the largest angular displacement of 1.1690 × 10^−3^ arcseconds. The LOS error in Table 2 is expressed in arcseconds (″), a geometric projection angular quantity that characterizes the apparent angular displacement of the infinite object in the object space after being imaged by the optical system, and its magnitude is determined by the optical projection ratio of the space telescope system.

The values in this table represent the percentage contribution of each mode to the total line-of-sight (LOS) error response. These results are obtained from frequency response analysis using the PSD excitations shown in Figure 8 (Y-wheel) and Figure 9 (Z-wheel).

### 3.2. Analysis of MTF Under Reaction Wheel Perturbation

Transient response analysis is capable of calculating the nodal displacement, nodal acceleration, and unit force of a structure subjected to time-varying excitation loads. This analysis is conducted for optical satellites to determine the positional changes in mirrors over time during the integration period. Given the high dimensionality of the entire satellite model, transient response analysis is executed using the direct method. The aforementioned analysis indicates that the 8th order mode significantly impacts line-of-sight (LOS) error; therefore, this mode serves as a case study for further examination. The oscillation period has been established at 0.03 s, leading to an excitation action duration set at 0.03 s.

Utilizing Nastran 2018 (MSC Software Corporation, Newport Beach, CA, USA) software, we analyze node deformation before processing with .bdf files and after processing with .pch files. Optical integration software Sig Fit is employed for fitting analysis, ultimately allowing us to calculate the rigid body displacement of each optical element in response to reaction wheel vibrations. Table 3 presents the rigid body displacements observed on each optical element surface under perturbations caused by reaction wheels. In this table, RB-TX/RB-TY/RB-TZ denote X/Y decentering and Z despace respectively; RB-RX/RB-RY/RB-RZ represent tilts about X/Y/Z axes correspondingly; SID1-SID5 indicate individual optical mirror surfaces. The tilt angles of the optical elements in Table 3 are expressed in radians (rad), a basic mechanical motion unit for describing the actual rotational inclination of the mirror surface. The numerical order of radians (10^−5^ rad level) is consistent with the arcseconds (10^−3^~10^−4^″ level) in Table 2 because 1 rad ≈ 2.06265 × 10^5^ arcseconds″, and the small tilt angle of the mirror (10^−5^ rad) is converted to the object-space LOS error (10^−4^″level) through the optical system’s magnification and projection effect, resulting in the same numerical order of the two physical quantities in different units.

**Table 3:** Average rigid body displacement and surface shape accuracy error of each reflecting surface (Unit: RB-TX/RB-TY/RB-TZ: mm; RB-RX/RB-RY/RB-RZ: rad; RMS: mm).

SID	RB-TX/mm	RB-TY/mm	RB-TZ/mm	RB-RX/rad	RB-RY/rad	RB-RZ/rad	RMS/mm
1	−1.8915 × 10^−3^	5.34401 × 10^−4^	−1.3281 × 10^−4^	1.7763 × 10^−5^	7.0699 × 10^−5^	−3.4549 × 10^−6^	3.03 × 10^−8^
2	−3.0537 × 10^−3^	8.07320 × 10^−4^	−1.2306 × 10^−4^	1.4336 × 10^−5^	6.5377 × 10^−5^	−3.0411 × 10^−6^	1.43 × 10^−8^
3	−1.3678 × 10^−3^	3.92634 × 10^−4^	−1.3693 × 10^−4^	2.6374 × 10^−5^	7.8048 × 10^−5^	−3.4999 × 10^−6^	2.96 × 10^−9^
4	−1.4117 × 10^−3^	3.8695 × 10^−4^	−2.7696 × 10^−4^	2.5965 × 10^−5^	7.0564 × 10^−5^	1.0752 × 10^−6^	2.77 × 10^−9^
5	−1.3971 × 10^−3^	4.4908 × 10^−4^	−7.5691 × 10^−4^	3.4412 × 10^−5^	7.6242 × 10^−5^	−6.2203 × 10^−6^	1.47 × 10^−9^

**Figure 10:**
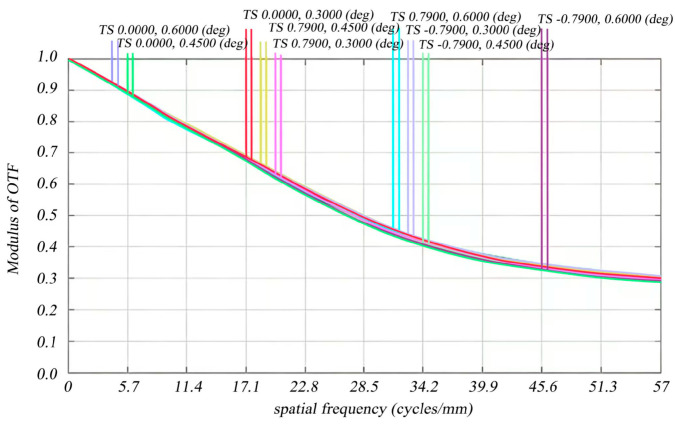
Initial design MTF curves for different fields of view at the Nyquist frequency (57 lp/mm).

**Figure 11:**
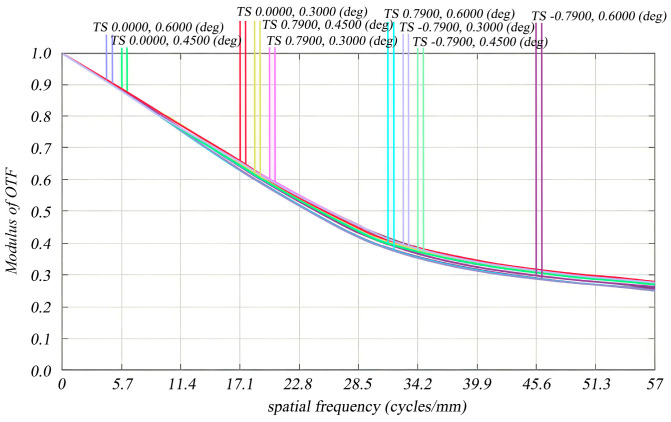
MTF curves of the optical system under reaction wheel disturbance for different fields of view.

Sig Fit generates a .zpl file that can be seamlessly imported into the ZEMAX macro file, facilitating a clear and visual representation of the modulation transfer function (MTF) degradation in the optical imaging system when the reflector mirror undergoes rigid body displacement and deformation due to reaction wheel perturbations. The intrinsic mechanism of MTF attenuation is closely related to the rigid body motion (translation/tilt) and micro-scale surface shape deformation of optical reflectors induced by reaction wheel micro-vibrations (Table 3): on the one hand, the micro-displacement (10^−3^ mm level) and micro-tilt (10^−5^ rad level) of the mirror change the ideal imaging position of the optical system, leading to the lateral jitter of the image point on the detector plane and the reduction in the spatial resolution of the optical system; on the other hand, the micro-scale surface shape error (10^−9^ mm level RMS) of the mirror causes the wavefront distortion of the incident light, which weakens the contrast transfer ability of the optical system for different spatial frequencies. In addition, the 8th and 9th order dominant modes (34.98 Hz/38.42 Hz) that contribute most to the LOS error further amplify the above optical aberrations, resulting in the most significant MTF attenuation in the high spatial frequency band (57 lp/mm) and the off-axis large field of view (e.g., (0.7900, 0.6000)°). The outcomes of the optical simulation are summarized in Table 4. As demonstrated in Figure 10 and Figure 11 (the horizontal axis is labeled as spatial frequency (lp/mm), the vertical axis as MTF), it is evident that the MTF diminishes to 0.270596—approaching the design value—for each field of view before and after perturbation at a resolution of 57 lp/mm, particularly for the (0.0000, 0.6000)° field of view within the meridional plane. The influence of reaction wheel disturbances on image quality remains minimal; thus, the optical system preserves satisfactory image quality throughout these perturbations.

**Figure 12:**
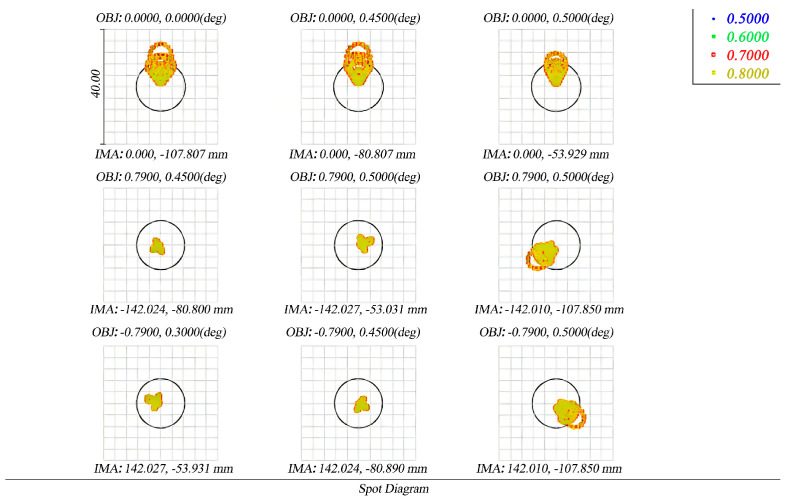
Initial design spot diagrams for different fields of view. The black circles in the diagrams represent the Airy spot (diffraction limit) of the optical system.

**Figure 13:**
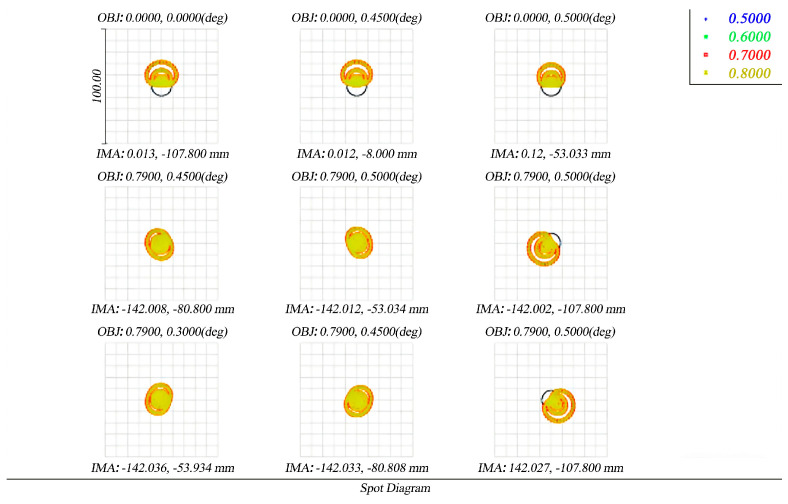
Spot diagram of optical system under reaction wheel disturbance. The black circles in the diagrams represent the Airy spot (diffraction limit) of the optical system.

The spot diagram before and after perturbation is presented in Figure 12 and Figure 13. Figure 12 shows the initial design, while Figure 13 shows the spot diagram under reaction wheel disturbance. The black circles in the diagram are the size of the Airy spot of the system. Taking the maximum diffuse spot rms diameter as an example, although the maximum diffuse spot rms diameter has slightly increased from 7.069 μm to 9.619 μm, the image point has some diffusion, but it is still close to an image element size (8.75 μm), which still meets the imaging requirements of the telescope.

## 4. Satellite Micro-Vibration Test

**Figure 14:**
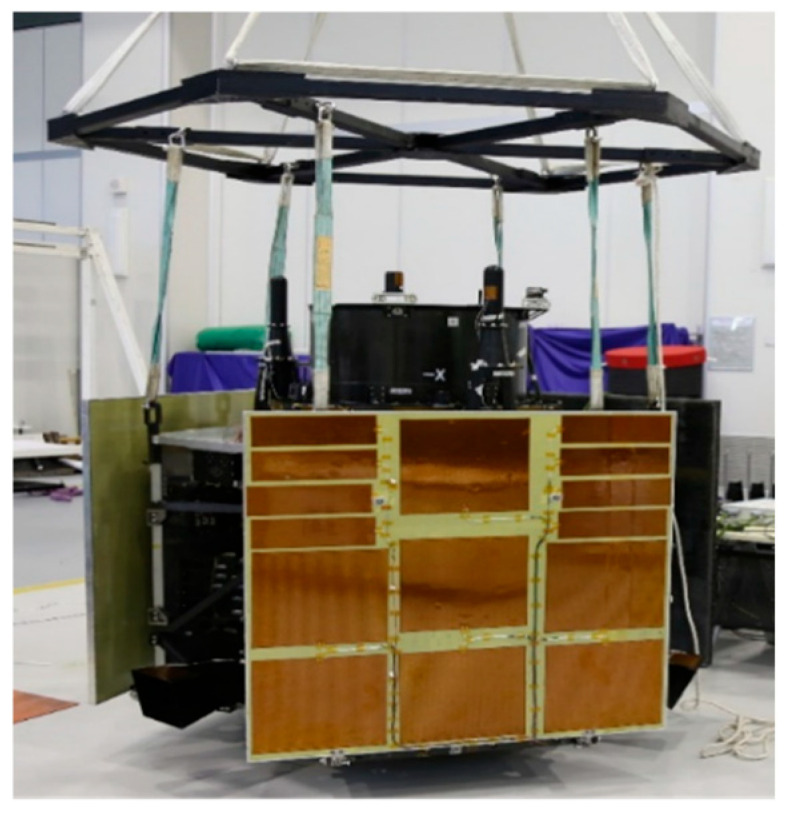
Satellite micro-vibration test site.

The satellite micro-vibration test was conducted in a specialized micro-vibration testing laboratory. The satellite was suspended vertically to simulate its orbital state, with the gyroscope mounted on the satellite’s space telescope. This setup aimed to measure the flutter of the reaction wheel during normal operation and to assess the angular displacement generated by objects in the optical telescope’s field of view. The sampling frequency for this test was set at 1000 Hz. Initially, background noise measurements were taken. According to the actual in-orbit control design strategy for the reaction wheel, measurements were recorded at various speeds: 1200 rpm, 1550 rpm, and 2078 rpm for the *Z*-axis reaction wheel under different satellite states. Corresponding data from the fiber optic gyro were also documented. Subsequently, after halting operations of the *Z*-axis reaction wheel, acceleration tests were performed on the *Y*-axis reaction wheel up to 600 rpm; data collection continued until it ceased functioning. Finally, both wheels were accelerated again—specifically, bringing the *Z*-axis reaction wheel up to 1200 rpm while maintaining *Y*-axis speed at 600 rpm. The detailed process of this micro-vibration test is illustrated in Figure 14.

**Figure 15:**
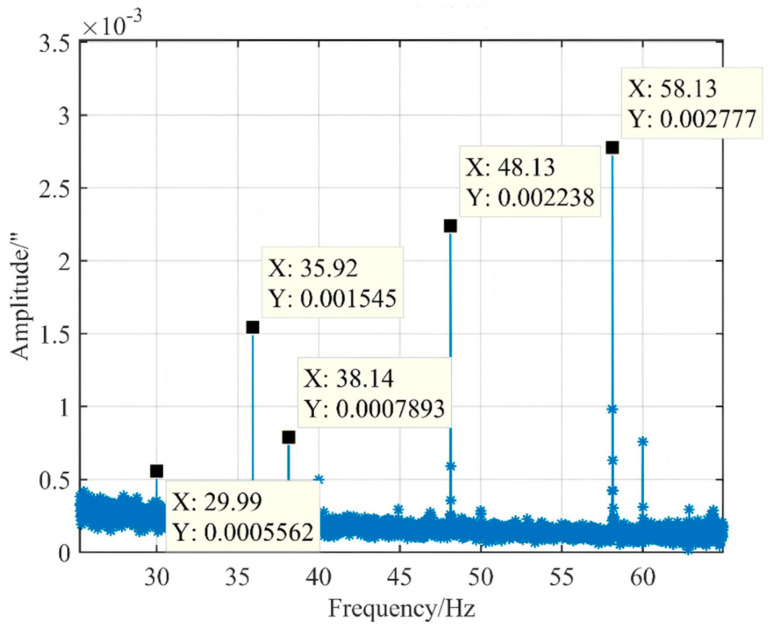
Space angular displacement of objects in the suspended state of the satellite.

The 8th and 9th order modes of the satellite exert a significant influence on the line-of-sight (LOS) error; therefore, we concentrate on the apparent axis error induced by the optical telescope in object space for these specific modes. As illustrated in Figure 15, when the satellite is in a suspended state, the reaction wheel speed along the *Z*-axis is set at 1200 rpm, while that along the *Y*-axis is maintained at 600 rpm. This configuration corresponds to an angular displacement of 0.001545 arc seconds for the 8th-order mode and 0.0007893 arc seconds for the 9th-order mode. The relative errors associated with these displacements are calculated to be approximately 9.34% and 6.52%, respectively. The comparison of MTF before and after perturbation for each field of view is presented in Table 5. It is tentatively concluded that micro-vibrations will not significantly degrade the quality of remote sensing images acquired in orbit, and that simulation analyses possess a certain degree of accuracy.

## 5. Conclusions

In micro-vibration analysis, reflectors are typically treated as rigid bodies, with the effects of mirror deformation on the optical system often overlooked. The opto-mechanical integrated analysis method presented in this paper enables a comprehensive assessment of how mirror rigid body displacement and deformation influence the imaging quality of the optical system under micro-vibrations induced by reaction wheel disturbances. This approach allows for an evaluation of the optical system’s performance during the design phase, ensuring that results from satellite micro-vibration analyses more accurately reflect real engineering conditions, and provides a high-precision micro-disturbance analysis framework for the design and optimization of micro/nano-scale precision optical systems. Quantitative results show that the 8th mode contributes 53.7% of the total LOS error, with a maximum angular displacement of 1.1690 × 10^−3^ arc seconds. The MTF degradation at the Nyquist frequency (57 lp/mm) is within 0.03 across all fields of view, and the relative errors between simulation and experiment are 9.34% and 6.52% for the two dominant modes. These findings can serve as direct quantitative references for the structural layout optimization and vibration isolation design of space optical satellites. More importantly, the proposed optomechanical integrated analysis framework, characterized by high precision in quantifying micro-displacements (on the order of 10^−3^ mm) and micro-angular displacements (on the order of 10^−5^ rad), is highly suitable for performance analysis of micro/nano-scale precision optical systems sensitive to micro-vibrations (e.g., micro-opto-electro-mechanical systems (MOEMS), micro-lithography objectives, micro-optical sensors). It addresses the methodological gap in optomechanical coupling analysis of micro-vibration effects for micro/nano-optical devices and holds significant engineering guidance value for the development of micro/nano-optical systems, near-eye display devices, and related applications in advanced manufacturing, electronic system engineering, and other relevant fields.

## Figures and Tables

**Table 1:** Contributions of each order mode to line-of-sight. The 4th–6th order modes with 0.00 Hz represent the rigid-body modes of the system (zero-frequency rigid-body motion).

Mode	Fre (Hz)	LI-TX (%)	LI-TY (%)	LI-TV (%)	LI-RX (%)	LI-RY (%)	LI-RV (%)	LO-RX (%)	LO-RY (%)	LO-RV (%)
4	0.00	0.006	0.730	0.672	0.685	0.007	0.305	0.730	0.006	0.332
5	0.00	0.003	0.024	0.022	0.034	0.001	0.015	0.024	0.003	0.013
6	0.00	0.011	0.106	0.098	0.122	0.012	0.060	0.106	0.011	0.054
7	29.62	0.003	0.001	0.001	0.001	0.003	0.002	0.001	0.003	0.002
8	34.98	51.726	56.196	55.834	57.302	51.465	54.025	56.196	51.726	53.737
9	38.42	34.530	39.548	39.141	38.405	34.586	36.261	39.548	34.530	36.788
10	41.18	0.000	0.001	0.001	0.001	0.000	0.000	0.001	0.000	0.001

**Table 2:** Line-of-sight error in object space (Unit: arcseconds, ″).

Mode	Fre/Hz	LO-RX/″	LO-RY/″	LO-RV/″
4	0.00	8.6704 × 10^−4^	8.5921 × 10^−5^	8.7129 × 10^−4^
5	0.00	8.9274 × 10^−5^	3.7313 × 10^−5^	9.6758 × 10^−5^
6	0.00	−4.0726 × 10^−4^	−1.4674 × 10^−4^	4.3289 × 10^−4^
7	29.62	4.3027 × 10^−4^	−3.1223 × 10^−4^	5.3162 × 10^−4^
8	34.98	7.4244 × 10^−4^	9.0296 × 10^−4^	1.1690 × 10^−3^
9	38.42	7.3921 × 10^−4^	−4.0069 × 10^−4^	8.4082 × 10^−4^
10	41.18	−1.8000 × 10^−4^	−1.6322 × 10^−4^	2.4541 × 10^−4^

**Table 4:** Comparison of MTF before and after perturbation at the Nyquist frequency for each field of view.

Field of View (°)	Designing MTF (Meridian)	MTF After Perturbation (Meridian)	Designing MTF (Arc Vector)	MTF After Perturbation (Arc Vector)
0.0000, 0.6000	0.299048	0.270596	0.292877	0.268685
0.0000, 0.4500	0.288669	0.272092	0.292646	0.270227
0.0000, 0.3000	0.293952	0.276456	0.296416	0.275490
0.7900, 0.4500	0.302077	0.272290	0.302067	0.263810
0.7900, 0.3000	0.301747	0.274945	0.301605	0.268758
0.7900, 0.6000	0.293102	0.258013	0.292625	0.249748
−0.7900, 0.3000	0.301747	0.274511	0.301605	0.268308
−0.7900, 0.4500	0.302077	0.271841	0.302067	0.263329
−0.7900, 0.6000	0.293102	0.257571	0.292625	0.249276

**Table 5:** Comparison of LOS angular displacement test and simulation results of the whole satellite.

Operating Conditions	Mode	Simulation Outcomes (″)	Experimental Results (″)	Relative Deviation *α*
The *Z*-axis reaction wheel rotates at 1200 rpm, and the *Y*-axis reaction wheel rotates at 600 rpm.	8	0.001690	0.001545	9.34%
	9	0.0008408	0.0007893	6.52%

## Data Availability

The data presented in this study are available on request from the corresponding author.
